# Community‐Based Participatory Research to Promote Social Inclusion in a Rural Town in Japan: Process Evaluation of a Pre‐COVID‐19 Collaboration Among Residents and Professionals

**DOI:** 10.1002/jgf2.70179

**Published:** 2026-09-17

**Authors:** Daisuke Son, Akira Matsushita, Michinori Tanaka, Mina Ueharu, Yusuke Yamauchi, Kunihiro Tsujikawa, Shoko Uetsuki, Naoko Tateishi, Masako Nishi, Akiko Ichii, Suhkye Shin, Naoki Sugawara

**Affiliations:** ^1^ Department of Community‐Based Family Medicine, Faculty of Medicine Tottori University Yonago Tottori Japan; ^2^ Department of Medical Education Studies, International Research Center for Medical Education, Graduate School of Medicine The University of Tokyo Tokyo Japan; ^3^ Nagi Family Clinic, Tsuyama Family Clinic, Yunogo Family Clinic, Family Practice Centre of Okayama Okayama Japan; ^4^ Okayama Psychiatric Medical Center Okayama Japan; ^5^ Morimoto Home Medical Care Clinic Matsuyama Ehime Japan; ^6^ Tsuyama Family Clinic, Family Practice Centre of Okayama Tsuyama Okayama Japan; ^7^ Nagi Town Office Okayama Japan; ^8^ Minna no Ouchi “Pokkappoka” Okayama Japan; ^9^ Connecting Communities Research Institute Okayama Japan; ^10^ Seinendan Theatre Company Toyooka Hyogo Japan

**Keywords:** community‐based participatory research, creative health, Japan, rural health, social inclusion

## Abstract

**Background:**

Rural communities face aging, depopulation, shrinking resources, and increasing social isolation. This study examined how a Community‐Based Participatory Research (CBPR) partnership was formed and negotiated in rural Japan, focusing on collaborative decision‐making and arts‐based engagement as a foundation for social inclusion.

**Methods:**

A qualitative descriptive study was conducted in Nagi Town, Okayama Prefecture, Japan. Between February 2018 and January 2020, eight CBPR meetings were held with 13 participants, including family physicians, public health nurses, community‐based participants, and theater professionals. Audio‐recorded discussions, field notes, and documents were analyzed thematically using Braun and Clarke's framework, focusing on partnership formation, equality and representation, arts‐based knowledge generation, decision‐making, and implementation challenges during the pre‐action phase.

**Results:**

Four cross‐cutting themes were identified: equality and mutual learning, discovery of hidden community strengths, arts as a bridge for dialogue and participation, and fragility and resilience of collaborative action planning. Collaboration evolved from facilitator‐led learning to shared local interpretation and distributed action planning. Two working groups explored ways to identify socially isolated residents and designed art‐ and memory‐based events. The “*Choi‐waru* Reunion”, using local photographs and films, created opportunities for intergenerational dialogue. The planned participatory theater workshop involving persons with disabilities or experiences of social withdrawal was canceled because of COVID‐19, but the preceding process supported co‐learning, creative planning, and cross‐sector relationships.

**Conclusions:**

CBPR combined with arts‐based engagement may support relationship‐building, shared reflection, and collaborative planning in rural communities. Rather than demonstrating intervention effectiveness, this study highlights preparatory processes as a foundation for future social inclusion efforts.

## Graphical abstract

This study illustrates how Community‐Based Participatory Research and arts‐based engagement supported a preparatory process for social inclusion in a rural Japanese town. Rather than demonstrating intervention effectiveness, the process fostered dialogue, creativity, and cross‐sector collaboration as a foundation for future community action.

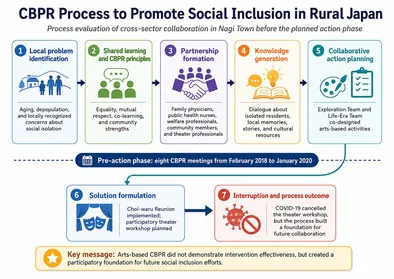

## 1 Introduction

Rural communities worldwide are facing complex challenges associated with population aging, depopulation, social isolation, and shrinking health and welfare resources [1, 2]. These challenges cannot be addressed by clinical services or formal welfare systems alone, because they are deeply embedded in local relationships, community participation, cultural practices, and everyday forms of mutual support [2]. Internationally, Community‐Based Participatory Research (CBPR) has been used to address such complex public health issues by bringing together researchers, professionals, and community members to identify local concerns, generate knowledge, and develop contextually meaningful actions [3, 4]. However, less is known about how CBPR partnerships are formed in small rural communities outside Western contexts, or how arts‐ and culture‐based engagement can contribute to social inclusion during the preparatory phase of participatory action.

Japan's rural municipalities face multiple, intertwined challenges including population aging, depopulation, and widening social isolation. As of 2024, more than 30% of the national population is aged 65 years or older, with the proportion exceeding 40% in some rural prefectures [2]. In such settings, medical and welfare professionals encounter difficulties reaching residents who are socially withdrawn, such as older men, persons with disabilities, or those experiencing “hikikomori” (social withdrawal) [5, 6]. These issues are not only medical or welfare concerns but also cultural and relational ones that call for community‐driven, interdisciplinary responses [5, 7].

Nagi Town, a mountainous municipality in northern Okayama Prefecture with approximately 5000 residents, offers an instructive case for examining these issues in a rural Japanese context. In 2020, the town had a population of 5578, a population density of 80.2 persons/km^2^, and an aging rate of 35.4%, which was higher than the national average of 28.6%. Although precise municipality‐level epidemiological data on social isolation were not available, local practitioners had identified social isolation among older adults, persons with disabilities, and individuals experiencing social withdrawal as a practical challenge that could not be fully addressed through conventional medical or welfare services alone. At the same time, the town had implemented community‐building and cultural revitalization initiatives. This combination of demographic vulnerability and existing community resources made Nagi Town a suitable setting for exploring CBPR as a means of fostering dialogue, empowerment, and mutual learning among residents and professionals.

CBPR has gained international recognition as a framework for equitable collaboration between researchers and communities [3, 4]. Informed by Paulo Freire's *Pedagogy of the Oppressed* [8], CBPR is grounded in the principles of empowerment, conscientization, and praxis, where reflection and action are mutually reinforcing. Rather than positioning communities as passive subjects, CBPR involves them as co‐researchers who collectively identify problems, generate knowledge, and implement solutions. Over the past two decades, CBPR has been applied to diverse public health issues worldwide, such as chronic disease prevention [3], environmental justice [9], and mental health promotion [10].

Arts‐based approaches offer a complementary pathway for CBPR because they can create accessible forms of participation that do not rely solely on verbal discussion, expert knowledge, or service‐based interaction. Creative Health and arts‐in‐health literature suggest that artistic and cultural engagement can promote social connectedness, emotional expression, identity formation, and well‐being by providing shared experiences through which people can tell stories, evoke memories, and recognize one another's capacities [11, 12, 13]. These features are particularly relevant to social inclusion, because socially isolated residents may find it difficult to participate in conventional meetings, health education programs, or welfare services. In this sense, photographs, films, songs, and theater can function as participatory media that support dialogue, memory‐sharing, and relationship‐building across generations and social positions.

In Japan, however, CBPR remains relatively underdeveloped compared with Western contexts. While community engagement has long existed through public health nursing and *minsei‐iin*, or civil welfare commissioners [14], formal research frameworks that ensure equitable partnership are rare. Most Japanese studies labeled “participatory” focus on health education, needs assessment, or service improvement led primarily by professionals [15]. Genuine co‐production, where residents, practitioners, and researchers share decision‐making authority, has been less frequently reported. Moreover, few examples have integrated cultural and artistic dimensions into CBPR, particularly in non‐Western rural contexts. In this study, art was conceptualized not as a therapeutic add‐on, but as a participatory medium through which residents and professionals could share memories, recognize local strengths, and collaboratively imagine inclusive forms of community action.

Despite this growing body of work, three gaps remain in the literature. First, most CBPR studies report implemented interventions and their outcomes, while less attention has been paid to the preparatory phase in which partnerships are formed, roles are negotiated, and locally meaningful actions are co‐designed. Second, although rural communities are often described as resource‐limited settings, few studies have examined how professional authority, local relationships, and administrative structures shape the development of participatory partnerships in small rural municipalities. Third, while arts‐based and creative health approaches have been increasingly discussed in relation to well‐being and social connectedness, their role within CBPR as a medium for dialogue, memory‐sharing, and social inclusion remains underexplored, particularly in non‐Western rural contexts.

The present study situates itself at this intersection between CBPR and arts‐based community health. The broader collaborative project was initiated in Nagi Town in 2017, with preparatory activities preceding the formal CBPR meetings; the eight meetings analyzed in the present study were conducted between February 2018 and January 2020. During this period, residents, welfare volunteers, healthcare professionals, and arts practitioners worked together to address local health challenges. Rather than implementing a predetermined intervention, the project emphasized partnership building, dialogical reflection, and creative planning. Through the eight CBPR meetings, participants explored issues of social isolation, developed new communication tools, and ultimately planned an inclusive theater workshop in which residents with disabilities or experiences of social withdrawal would play central roles. Although the performance itself was canceled due to the onset of the COVID‐19 pandemic, the preceding process provides insight into rural partnership formation and the potential of arts‐based CBPR to build a foundation for social inclusion.

This study aimed to examine how a CBPR partnership was formed and negotiated among healthcare and welfare professionals, arts practitioners, and community members during the preparatory phase of a rural Japanese project for social inclusion. Using audio‐recorded meeting discussions, field notes, and project documents, we conducted a thematic analysis focusing on partnership formation, negotiation of equality and representation, arts‐based knowledge generation, collaborative decision‐making, and challenges to implementation.

By examining this formative process, the study contributes to international CBPR literature by showing how participatory partnerships can be initiated before formal intervention implementation, and how arts‐ and memory‐based engagement may support dialogue, relationship‐building, and collaborative action planning in a non‐Western rural context.

## 2 Methods

### 2.1 Study Design

This study employed a qualitative descriptive design framed within the principles of CBPR [4]. CBPR was chosen as the guiding methodology because it emphasizes collaboration between researchers and community members in identifying local issues, generating knowledge, and planning actions. The study was designed as a process evaluation of the preparatory or pre‐action phase of a larger CBPR initiative, rather than as an evaluation of intervention outcomes. The broader project was initiated in 2017 with preparatory activities, while the eight structured CBPR meetings analyzed in this study were held between February 2018 and January 2020. Each meeting lasted approximately 2 h and included educational and participatory components.

This study was guided by a pragmatic CBPR orientation [3, 4, 16]. This orientation was appropriate because the project examined a real‐world community process while also supporting the development of locally feasible action plans. Freirean critical pedagogy informed our attention to empowerment, conscientization, praxis, and power relationships among researchers, professionals, and residents [8]. A social constructivist perspective also informed the analysis of how participants developed shared understandings through dialogue, storytelling, memory work, and arts‐based activities [17]. These perspectives were used as sensitizing frameworks during analysis, particularly when examining how participation, equality, power relationships, and shared meanings appeared in the meeting data.

The project group consisted of family physicians, public health nurses, a social worker, community‐based participants, and theater professionals. The first author, an academic family physician with prior experience in CBPR, participated in all eight meetings and served as the main academic facilitator. His roles included introducing CBPR principles, preparing meeting agendas, supporting facilitation, preparing field notes and analytic memos, and leading the initial data analysis. Other members contributed clinical, welfare, artistic, and community‐based expertise, although they did not necessarily have prior formal experience in CBPR. Each meeting included three procedural components: a short introduction to CBPR concepts by the first author, facilitated discussion of local health and welfare concerns, and group planning or review of possible actions.

At the beginning of the project and repeatedly during subsequent meetings, the first author introduced and discussed the nine principles of CBPR proposed by Israel and colleagues and further elaborated in subsequent CBPR literature [3, 4]. These principles were: (1) recognizing the community as a unit of identity; (2) building on strengths and resources within the community; (3) facilitating collaborative, equitable partnerships in all phases of research; (4) promoting co‐learning and capacity building among all partners; (5) integrating knowledge and action for the mutual benefit of all partners; (6) emphasizing a cyclical and iterative process; (7) addressing health from both positive and ecological perspectives; (8) disseminating findings and knowledge to all partners; and (9) involving a long‐term process and commitment to sustainability. These principles were introduced as part of the meeting materials and were used to structure discussion, reflection, and planning during the meetings.

### 2.2 Setting and Participants

The study was conducted in Nagi Town, a small mountainous municipality in northern Okayama Prefecture, western Japan. Nagi Town was selected as the study site for three reasons. First, it represents a typical rural Japanese community facing population aging and gradual depopulation while still maintaining active local health, welfare, and community‐building activities. According to regional statistics, the town had a population of 5578 in 2020, a population decline of 5.55% from 2015 to 2020, a population density of 80.2 persons/km^2^, and an aging rate of 35.4%, which was higher than the national average of 28.6% in 2020. Second, local practitioners had identified social isolation among older adults, persons with disabilities, and socially withdrawn individuals as a practical challenge that could not be fully addressed through conventional medical or welfare services alone. Third, Nagi Town had a pre‐existing foundation of community engagement and cultural activities, including art‐ and memory‐based local initiatives, making it a suitable setting for exploring the feasibility of CBPR combined with creative health approaches.

Although precise municipality‐level epidemiological data on social isolation were not available, local administrative documents anticipated an increase in older adults living alone and older couple households and emphasized the need to promote social participation and community‐based support for older residents. In this study, therefore, “social isolation” was not defined as a statistically measured prevalence but as a locally recognized practical concern identified through discussions among healthcare professionals, welfare workers, community volunteers, and residents.

Participants were initially recruited through a purposive process led by a family physician actively engaged in community development in Nagi Town. In response to the physician's invitation, family physicians, public health nurses, a social worker, community‐based participants, and theater professionals joined the project. As the project developed, additional participants, including colleagues and community members interested in the activities, were introduced through snowball sampling. Because the initial aim was to establish a cross‐sector partnership and explore locally feasible approaches to social isolation, socially isolated residents themselves were not systematically recruited during the early meetings. Their perspectives were reflected mainly indirectly through public health nurses, welfare professionals, community volunteers, and family‐related narratives discussed during the process.

For this study, participants were defined as individuals who attended at least one of the eight CBPR meetings and consented to the use of meeting data for research purposes. A total of 13 individuals participated in at least one meeting. Attendance at each meeting ranged from 8 to 11 participants, with approximately 10 core members attending regularly. Participants consisted of six family physicians, two public health nurses, one social worker, one community volunteer, one community activist, and two theater professionals. Participant characteristics, including primary role or occupation, sector, length of involvement in community health, welfare, or other community activities, and meeting attendance, are summarized in Table 1.

**TABLE 1:** Characteristics of participants in the CBPR meetings.

Participant ID	Primary role in the project	Professional/community background	Sector	Length of involvement in community health, welfare, or community activities	Meeting attendance
P1	Family physician/academic facilitator/researcher	Family medicine, medical education, CBPR	Healthcare/academia/research	> 10 years	8 meetings
P2	Family physician/local practitioner	Family medicine, community health	Healthcare/academia	> 10 years	8 meetings
P3	Family physician/practitioner	Family medicine, mental health	Healthcare	> 5 years	4 meetings
P4	Family physician/practitioner	Family medicine, local primary care	Healthcare	> 5 years	4 meetings
P5	Family physician/practitioner	Family medicine, local primary care	Healthcare	> 5 years	6 meetings
P6	Family physician/practitioner	Family medicine, local primary care	Healthcare	> 5 years	6 meetings
P7	Public health nurse	Public health, municipal health/welfare services	Health/welfare administration	> 10 years	7 meetings
P8	Public health nurse	Public health, municipal health/welfare services	Health/welfare administration	> 10 years	5 meetings
P9	Social worker	Social welfare, community support	Welfare	1–5 years	3 meetings
P10	Community volunteer	Community support, caregiving/welfare‐related volunteer activity	Community	> 10 years	6 meetings
P11	Community activist	Community development, resident engagement	Community/nonprofit	> 10 years	5 meetings
P12	Theater professional	Theater practice, community arts	Arts/culture	> 5 years	5 meetings
P13	Theater professional	Participatory theater, community arts	Arts/culture	> 10 years	5 meetings

*Note:* Participants were categorized according to their primary role in the project. Because several participants had multiple professional and community roles, these categories describe their main contribution to the CBPR process and are not intended to represent mutually exclusive identities. Age group and sex were reported in aggregated form to protect anonymity in this small rural setting.

Because this project was conducted as a participatory community initiative, membership remained intentionally open and fluid rather than fixed. The unit of analysis in this study was therefore the collaborative process across the eight meetings and related documents, rather than individual interviews with a predetermined sample. Participants were invited to contribute their professional expertise, local knowledge, and practice‐based or community‐based experiences to the discussions. Although the project aimed to promote equal participation, we recognized that differences in professional status, community roles, and prior relationships could influence the interactional dynamics within the meetings; this issue was considered during analysis and is addressed in the Discussion.

### 2.3 Researcher Roles and Reflexivity

The first author was an academic family physician with prior formal experience in CBPR and qualitative research. He participated in all eight meetings and served as the main academic facilitator. His roles included introducing CBPR principles, supporting meeting design and facilitation, preparing field notes and analytic memos, and leading the initial data analysis. The second author was a family physician actively engaged in community development in Nagi Town and played a central role in recruiting participants, coordinating local stakeholders, and interpreting the local context. Other members contributed clinical, public health, welfare, community‐based, and artistic perspectives to the interpretation of the process.

Because several researchers were also participants in the CBPR process, we recognized that professional status, prior relationships, and the authority of physicians or municipal professionals could influence meeting interactions and data interpretation. To address this, the first author maintained reflexive memos after each meeting, documenting the atmosphere of discussions, emerging power dynamics, and his own positionality as both facilitator and researcher. During analysis, interpretations were discussed among members with different professional and community backgrounds to avoid relying solely on the academic researcher's perspective.

In particular, we did not assume that symbolic practices such as using “‐san” eliminated hierarchy. Instead, we treated such practices as data for examining how equality was attempted, negotiated, and only partially achieved within persistent professional and institutional power relations.

### 2.4 Data Collection

Multiple sources of data were used to capture both the process and content of collaboration. All eight meetings were audio‐recorded, yielding approximately 13 h of recordings, which were transcribed verbatim in Japanese. Field notes were written by the academic facilitator immediately after each CBPR meeting to document contextual details such as atmosphere, non‐verbal interactions, and reflexive impressions. Additional documentary materials produced during the project—such as flyers, drafts of community questionnaires, photographs, event programs, and anonymized summaries of outreach‐related discussions—were collected to supplement the transcripts. Although the Exploration Team discussed residents who might be socially isolated, personally identifiable lists of residents were not retained or analyzed as research data. When such information was discussed during meetings, names, addresses, and other identifying details were removed from transcripts and field notes, and the analysis used only anonymized or aggregated descriptions of the issues raised.

Although a fixed interview guide was not used, each meeting followed a semi‐structured agenda prepared by the facilitator. Each meeting began with a brief mini‐lecture introducing CBPR concepts such as partnership, empowerment, co‐learning, or evaluation, followed by group discussions and plenary reflections. The agenda typically included reflection on local concerns related to social isolation, discussion of possible actions, and sharing of ideas and next steps. Facilitators used open‐ended prompts to encourage participation and dialogue, such as: “What kinds of residents are difficult to reach through existing services?”, “What strengths or resources already exist in Nagi Town?”, “How can we create opportunities for residents who rarely participate in community activities?”, and “What forms of art, memory, or storytelling might help people connect?” These prompts were adapted flexibly according to the stage of the project and participants' emerging concerns.

The research dataset therefore consisted of verbatim transcripts, field notes, documentary materials, and analytic memos. To ensure accuracy, all transcripts were anonymized by removing names, addresses, and institutional identifiers. Analytic memos were kept after every meeting to record emerging topics, facilitation issues, possible power dynamics, and potential researcher biases. Interim summaries of findings were shared with participants for member checking, allowing them to confirm, modify, or expand the researchers' interpretations.

### 2.5 Data Analysis

The data were analyzed using thematic analysis following the six‐step framework proposed by Braun and Clarke [18]. The first author first read all transcripts, field notes, and documentary materials repeatedly to become familiar with the data. He then conducted initial coding of all meeting transcripts and field notes using NVivo 12 software (QSR International). Consistent with the study aim, the coding focused on observable processes within the meeting data related to partnership formation and negotiation during the preparatory phase. Specifically, the analysis examined how participants framed local problems, negotiated equality and representation, generated knowledge through dialogue and arts‐based activities, made collaborative decisions, and addressed tensions or challenges to implementation. Initial codes were grouped into broader candidate themes and iteratively refined.

The second author reviewed the initial codes and engaged in repeated discussions with the first author to refine and consolidate the coding framework, particularly in relation to the local context of Nagi Town and the development of the project. Emerging themes were then reviewed and discussed within the research team, which included members with clinical, public health, welfare, community‐based, and artistic backgrounds. These discussions were used to examine the coherence of the themes and compare interpretations across different professional and community perspectives.

To enhance trustworthiness, the team used triangulation of data sources, including meeting transcripts, field notes, and project documents, as well as investigator triangulation through repeated discussions among academic and community‐based members. Reflexive memos and an audit trail were used to document analytic decisions, emerging interpretations, and changes in the coding framework.

During coding and theme refinement, particular attention was paid to discrepant data and negative cases, including disagreements, hesitation, silence, uncertainty about the project's direction, and comments that challenged an overly smooth interpretation of consensus‐building. Such data were not treated as exceptions to be excluded, but as important indicators of how equality, participation, and collaborative decision‐making were negotiated in practice.

### 2.6 Ethical Considerations

Ethical approval for the study was granted by the Institutional Review Board of the Faculty of Medicine, University of Tokyo (Approval No. 11760). Because the research overlapped with ongoing community activities, special attention was paid to the ethical complexities of participatory work in small populations. At each meeting, participants received detailed oral and written explanations regarding the study's objectives, recording procedures, and data use. Written consent was obtained for interviews, and oral consent was accepted for group discussions where written signatures could compromise anonymity. When photographs or other visual materials were used for dissemination, additional consent was obtained from those depicted. When local professionals referred to residents who might be socially isolated, such information was managed within the usual professional and administrative context and was not copied or retained by the academic researchers as identifiable research data. Any references to specific individuals were anonymized during transcription and excluded from analysis.

## 3 Results

### 3.1 Overview

The Results are presented in two parts. First, we describe the chronological development of the CBPR process across the early, middle, and late phases to provide contextual understanding of how the project unfolded. Second, we present cross‐cutting thematic findings derived from thematic analysis. These themes do not simply correspond to the chronological stages of the meetings; rather, they capture recurring processes through which participants negotiated equality, participation, representation, and collaborative action across the entire project.

Across the eight CBPR meetings, collaboration evolved through three interrelated shifts. First, the process moved from facilitator‐led learning about CBPR principles to shared discussion of how these principles could be applied in Nagi Town. Second, participants gradually shifted from a professional problem‐identification frame toward a more appreciative and relational approach, in which socially isolated residents were discussed not only as people in need of support but also as people with hidden interests, memories, skills, and capacities. Third, collaboration moved from general dialogue to distributed action planning, as participants formed two working groups and began to use their different forms of expertise—clinical, welfare, community‐based, and artistic—to design locally meaningful activities.

Table 2 summarizes the eight CBPR meetings held between February 2018 and January 2020. During the process, two collaborative working groups were formed. One was the Exploration Team—nicknamed the “Tanken Hakken Hotto‐ken Team”—which focused on identifying residents who were socially isolated and exploring ways to reconnect them with the community. The other was the Life‐Era Team—referred to locally as the “Jinsei Jidai Team”—which sought to design events centered on art, film, and memory to stimulate intergenerational dialogue. These two groups represented different but complementary modes of collaboration: the Exploration Team drew mainly on professional and volunteer knowledge of residents who were difficult to reach, whereas the Life‐Era Team developed arts‐ and memory‐based spaces where residents could participate through stories, songs, photographs, and shared local history.

**TABLE 2:** Summary of the eight CBPR meetings conducted in Nagi Town (2018–2020).

Meeting	Date	Main discussion themes	Decisions/Key outcomes
1st meeting	February 25, 2018	Introduction to CBPR philosophy; equality among participants; exploring local health and social issues	Agreed on nine CBPR principles; established shared rule to address one another with “‐san” regardless of occupation; confirmed aim to tackle isolation using community strengths
2nd meeting	April 8, 2018	Brainstorming creative ways to engage socially isolated residents (older men, youth)	Proposed “Life‐Era” concept (karaoke, film screening); decided to form subgroups for further exploration
3rd meeting	June 24, 2018	Deepening partnership; linking art and health; identifying potential collaborators	Formed two working groups: “Exploration Team” to locate isolated residents and “Life‐Era Team” to design art‐based activities
4th meeting	August 18, 2018	Planning an intergenerational cultural event; discussion with film scholar from “Silver Cinema Paradise”	Decided to organize the town event “Choi‐waru Reunion—Let's Talk about the Showa Days”
5th meeting	January 14, 2019	Reflection on the Choi‐waru Reunion; evaluation of participation and community reactions	Recognized increased engagement of older men and intergenerational communication; agreed to continue with arts‐based approaches
6th meeting	August 25, 2019	Revisiting goals after administrative changes; idea for inclusive participatory theater	Agreed to plan a theater workshop involving persons with disabilities and those experiencing social withdrawal
7th meeting	November 4, 2019	Finalizing the theater project; scripts, roles, and rehearsal plans	Completed detailed structure for the participatory theater; scheduled rehearsals for early 2020
8th meeting	January 12, 2020	Refining the theater concept; discussion of “R's Story”, a real‐life narrative of social withdrawal	Decided on two‐part performance: (1) documentary interviews with R's parents, (2) dramatized “imagined future” of R; emphasized “a place for everyone” as the project's unifying theme; rehearsals planned for Feb 2020 (later canceled due to COVID‐19)

*Note:* Meetings were held approximately every few months, alternating between educational reflection and participatory planning. Data from all eight CBPR meetings informed thematic analysis in this study.

### 3.2 Early Phase (February–April 2018): Shared Learning and Equality

The first meeting served primarily as an introduction to the philosophy and methods of CBPR. The facilitator emphasized that this approach was “a research for improving practice, not research for its own sake”. Participants discussed how the nine CBPR principles could be translated into their local context, particularly the ideas of equitable partnership, co‐learning, building on community strengths, and linking reflection with action. They repeatedly returned to the themes of equality and mutual respect. One member proposed, “Even if our occupations differ, we should work side by side. Let's call each other ‘‐san’ instead of ‘doctor’ or ‘official’”. This discussion reflected a conscious attempt to flatten professional hierarchies and to construct a more horizontal mode of collaboration. However, this symbolic practice did not eliminate underlying power asymmetries. Because the project was initiated through a physician's invitation and the early meetings were facilitated mainly by physicians and an academic researcher, local residents and non‐professional participants sometimes responded to professionally framed agendas rather than setting topics independently. Field notes also suggested hesitation to express disagreement openly in discussions involving health or welfare issues. Thus, equality was not an achieved condition but an ongoing aspiration that had to be negotiated throughout the process. Members reflected on Paulo Freire's notion of *conscientization*—raising awareness through dialogue—and agreed to pursue “local problem‐solving rooted in community strengths”. This initial dialogue established a shared vocabulary of partnership that would guide subsequent activities.

At the second meeting, the focus shifted from theory to imagination. Participants brainstormed ways to “spotlight wonderful yet overlooked people” in their community, such as reclusive older men or younger residents quietly engaged in crafts. Out of this conversation emerged creative ideas including a “Life‐Era Karaoke”, where residents would share songs that shaped their youth, and a “Life‐Era Cinema”, in which people could screen favorite films and discuss memories associated with them. The tone of the meetings became increasingly lively, as laughter, anecdotes, and curiosity replaced initial hesitation. By the end of the spring of 2018, participants had built sufficient trust to begin experimenting with action‐oriented plans.

### 3.3 Middle Phase (June 2018–January 2019): Collaborative Action Planning

During the middle phase, collaboration became more distributed. Rather than relying mainly on the facilitator's explanation of CBPR, participants began to divide responsibilities according to their professional, community‐based, and artistic strengths. The Exploration Team drew on the knowledge of public health nurses, welfare professionals, and local volunteers to identify residents who were difficult to reach through existing services, while the Life‐Era Team translated earlier discussions about hidden strengths into a concrete cultural event.

The Exploration Team worked with civil welfare commissioners and local welfare volunteers to establish an information‐sharing process for residents who might otherwise remain unseen. Reporting forms were distributed during a joint training session attended by nearly 50 local welfare volunteers. Although the number of responses was limited, several cases of concern were identified, including older adults living alone and younger adults who had withdrawn from community life. During this process, participants also recognized the ambiguity of defining social withdrawal. One participant noted that some volunteers were unsure “how far we should go in defining someone as withdrawn”, suggesting that isolation was not a fixed category but a locally interpreted and relational concern.

At the same time, the Life‐Era Team advanced its plan to organize a cultural event that would bring together different generations through shared memories. Collaboration with a film scholar who operated “Silver Cinema Paradise”, a volunteer program screening classic movies in nursing homes, provided both inspiration and practical know‐how. Building on this connection, the team decided to host a town‐wide event titled “Choi‐waru Reunion—Let's Talk about the Showa Days”. Held on August 30, 2018, the event attracted approximately 50 residents, including several older men who rarely participated in public gatherings, as well as around 20 younger visitors such as students, community nurses, and local artists. As old photographs of Nagi's streets, buses, and everyday life were projected on screen, conversations emerged spontaneously between older residents and younger participants. Younger participants asked about unfamiliar scenes, while older residents explained local memories and histories. The gathering culminated in a collective singing of “Ringo no Uta”, which one participant described as a moment when “everyone was smiling and singing, and became one”. Encouraged by the response, staff at nursing homes and community centers later reused the slide show for reminiscence activities, extending the use of the materials beyond the original event.

### 3.4 Late Phase (August 2019–January 2020): Toward Inclusive Art and Pandemic Disruption

In the late phase, collaboration became more ambitious but also more fragile. Participants attempted to extend the arts‐based approach from reminiscence and intergenerational dialogue toward a participatory theater workshop involving people with disabilities or experiences of social withdrawal. At the same time, administrative transitions within the town disrupted the local initiative Nagi‐Kara and created uncertainty about how the project could continue. Rather than presenting this disruption only as an obstacle, participants discussed which elements could still be continued, including the Choi‐waru activities and the project for engaging people who tended to remain at home.

The theater plan developed through this process of adjustment. Participants did not limit participation to acting, but discussed multiple possible roles, including set‐making, lighting, reception, and ticket‐taking, so that people could be involved according to their interests and comfort levels. One participant captured this aspiration by saying, “We want those who have been watching from the corner to stand on stage”. By November 2019, the group had developed scripts, role assignments, and rehearsal plans. In January 2020, an additional meeting further refined the concept, focusing on the real‐life story of a young man living in social withdrawal (“R”) and his family. This discussion represented an attempt to bring the experiences of social withdrawal closer to the planning process, although these experiences were still mediated mainly through family narratives and the interpretations of professionals and community members rather than through R's direct participation.

Participants planned a two‐part performance: a documentary‐style film featuring interviews with R's parents, and a dramatized segment portraying “R's imagined future” as the owner of a used bookstore connecting with others through art and music. The theme “a place for everyone” symbolized theater as a safe communal space where participation could take different forms. Rehearsals were scheduled for February 2020, but the onset of COVID‐19 forced indefinite postponement. Thus, the collaboration was sustained not through uninterrupted implementation, but through repeated adjustments of goals, roles, and feasible forms of participation under changing local conditions.

### 3.5 Thematic Findings Across the CBPR Process

Thematic analysis identified four cross‐cutting process themes: (1) *equality and mutual learning*, (2) *discovery of hidden community strengths*, (3) *arts as a bridge for dialogue and participation*, and (4) *fragility and resilience of collaborative action planning*. These themes did not correspond directly to the early, middle, and late phases described above. Rather, they captured recurring patterns across the process, including participants' repeated attempts to interpret CBPR principles, negotiate the meaning of social isolation, adjust professional and community roles, and translate dialogue into feasible community action. Disagreements, hesitation, and uncertainty were treated not as exceptions but as important data showing how participation and ownership were negotiated in practice.

The first theme, *equality and mutual learning*, emerged from early discussions about how to create more horizontal relationships among researchers, professionals, artists, and community members. In the first meeting, the facilitator explained that CBPR was “not research for the sake of research”, but “an effort to improve practice”. When discussing equality, one participant interpreted it as “setting aside differences between occupations, or between residents and professionals, and moving forward together”. This led to a concrete exchange about whether members should address one another with *‐san* rather than occupational titles. Participants joked, “Should we call everyone ‘doctor’?” and “Either everyone is ‘doctor’ or everyone is ‘‐san’,” while another added, “I may say it without thinking.” These exchanges showed that equality was not achieved simply by declaration, but had to be repeatedly practiced and negotiated in everyday interaction.

The second theme, *discovery of hidden community strengths*, reflected a shift from viewing socially isolated residents only as “people in need of support” toward recognizing overlooked abilities, interests, and relationships. Participants described the aim as “finding hidden wonderful people, spotlighting what is wonderful about them, and helping them connect with people in the community”. Ideas such as “Life‐Era Karaoke” and “Life‐Era Cinema” emerged from this perspective, focusing less on performance skill than on the memories and life stories evoked through songs, films, and personal histories. A discussion about a resident's handmade wooden Gundam models also helped participants reframe withdrawal not simply as deficit or risk, but as a situation in which unrecognized interests and capacities might remain hidden. At the same time, participants critically questioned whether terms such as *hikikomori* or “discovery” might reproduce a one‐sided professional stance. One member noted that *hikikomori* already implied that “one should not be withdrawn”, while another suggested that “encounter” might better express respect for residents' own wishes. This discussion showed how participation was negotiated through language.

The third theme, *arts as a bridge for dialogue and participation*, emerged as participants explored non‐clinical forms of engagement through photographs, films, songs, memory, and performance. In discussing film‐based events, one participant observed that “rather than just talking”, it would be better to have “materials, photographs, or objects”. This idea was realized in the Choi‐waru Reunion, where old photographs of Nagi Town enabled older residents and younger participants to talk together about local memories. Participants described scenes in which younger people asked about unfamiliar images and older residents explained them, and the event ended with everyone singing “Ringo no Uta”. These accounts suggested that arts‐ and memory‐based materials functioned as mediating objects, allowing people to participate without being positioned primarily as patients, service users, or targets of welfare intervention.

The fourth theme, *fragility and resilience of collaborative action planning*, captured both the vulnerability of the project and participants' efforts to continue revising their plans. Practical tensions appeared repeatedly. Regarding the Choi‐waru activities, one participant reflected, “We must not make it staged, and we must not lead them too much”, while another summarized the difficulty of facilitation as “not pushing too much, but not leaving it alone either”. These comments showed that participation required careful adjustment: too much professional direction could undermine ownership, whereas too little support could leave ideas unrealized. The project was also affected by administrative change. When the local initiative Nagi‐Kara was discontinued, one participant described being unable to recover from the “Nagi‐Kara shock,” and others expressed anger and disappointment that the disruption occurred just as the project seemed to be moving forward. Yet collaboration continued through concrete adjustments: participants discussed “the parts that can be continued”, maintained the Choi‐waru activities, and redesigned the theater project so that people could participate not only as actors but also through set‐making, lighting, reception, and ticket‐taking. These examples indicated that collaboration was sustained not through uninterrupted implementation, but through repeated revision of goals, redistribution of roles, and flexible reconsideration of what participation could mean.

Across these themes, the CBPR process did not unfold as a smooth or linear consensus‐building process. Participants repeatedly confronted unresolved questions about representation, professional authority, administrative instability, and the difficulty of involving those least visible in the community. The voices of socially isolated residents themselves were not yet fully present in the early phase, and community members were largely reached through existing professional and volunteer networks. These tensions led the team to interpret participation not as a stable achievement, but as a fragile and negotiated process shaped by local relationships, power gradients, and changing institutional conditions.

## 4 Discussion

This study illustrates how the principles of CBPR were introduced and negotiated in a small rural town in Japan. Although the action phase—an inclusive theater performance—was canceled due to the COVID‐19 pandemic, the preceding process generated important process‐level developments: attempts to build more horizontal partnerships, the co‐creation of culturally grounded ideas, and the development of shared reflection and collaborative planning. Through repeated dialogue across eight meetings, residents and professionals jointly reframed their understanding of “research” as a relational and reflective practice rather than a technical procedure. The project suggests that the process of collaboration itself can be a meaningful component of CBPR, even before intervention outcomes are demonstrated.

Japanese public health activities have long relied on top‐down administrative programs led by government and professionals [19]. While this structure has supported national health improvements, it can also limit local agency and reinforce professional authority [19]. Against this background, the Nagi project attempted to create a more horizontal mode of collaboration through repeated discussion of CBPR principles, co‐learning, and symbolic practices such as addressing one another as “‐san” regardless of occupation. This practice represented a culturally meaningful effort to reinterpret CBPR through local values of respect, harmony, and collective responsibility, and it resonated with Freire's concept of dialogue as a mutual act of recognition as well as Japan's traditions of *chiiki zukuri* (community making) and *kyōsei* (co‐living) [20, 21].

At the same time, this symbolic effort did not eliminate deeper power asymmetries. The project was initiated through a physician's invitation, and physicians, municipal professionals, and an academic researcher inevitably held social and institutional authority in the rural setting. Their presence may have influenced which issues were recognized as legitimate, how agendas were framed, and how freely residents or community volunteers felt able to disagree. We therefore interpret equality in this CBPR process not as an achieved condition, but as an aspiration that required continuous negotiation. Although the team attempted to mitigate these asymmetries through repeated reflection on CBPR principles and arts‐ and memory‐based activities that allowed different forms of knowledge to emerge, these strategies only partially addressed professional authority. Future CBPR projects should incorporate more explicit mechanisms for shared agenda setting, facilitation by non‐professional community members, and earlier involvement of those most affected by the issues under study.

A distinctive feature of this initiative was the integration of artistic activities—photography, film, and theater—into the participatory process. These forms of expression acted as relational infrastructure, providing emotionally safe spaces for residents to connect across generations and social boundaries. The “Choi‐waru Reunion”, for instance, transformed nostalgia into intergenerational dialogue, as elderly participants shared memories while younger residents rediscovered local history. Such art‐based encounters paralleled findings from international “Arts in Health” research, which links aesthetic participation to enhanced social connectedness and well‐being [12, 13, 22]. In this project, art was not a therapeutic add‐on but a method of inquiry and dialogue. This convergence between CBPR and “Creative Health” aligns with recent Japanese discussions on cultural prescribing, in which creative and cultural engagement are integrated into public health and primary care [11, 12].

Several challenges emerged during the project. Administrative and political transitions within the town disrupted continuity, highlighting the need for institutional support and long‐term anchoring of CBPR initiatives. Sustaining voluntary participation also required significant time, emotional energy, and facilitation. Meetings themselves became spaces of mutual care where members navigated differences in pace, motivation, and expectations. Finally, the onset of the COVID‐19 pandemic exposed the vulnerability of community projects to external crises. Yet, despite the interruption, some relationships and principles developed through the CBPR process continued to inform subsequent welfare outreach and educational activities. This suggests that one important process‐level outcome of the project lay in changes in participants' orientations toward collaboration, rather than in tangible outputs alone.

For rural primary‐care practitioners, CBPR offers a framework to address the social determinants of health beyond the clinic. By involving residents as co‐researchers, clinicians can gain a deeper understanding of local realities such as isolation, aging, and disability. The integration of arts and participatory dialogue expands the concept of “social prescribing” within Japan's community‐based integrated care system (*chiiki‐hōkatsu care*) [23, 24]. Physicians and public health nurses can act as facilitators of collective meaning‐making rather than sole providers of interventions. This reframing supports a more humane and sustainable model of rural health promotion, where care is produced through shared creativity and trust.

The study's strengths include rich qualitative data gathered over 2 years and the direct involvement of community members in both data generation and interpretation, which enhanced credibility and contextual validity. The project also contributes to the limited body of literature on rural CBPR in Japan by illustrating how participatory and artistic methods can reinforce each other.

This study has several limitations. First, it focused on a single rural community, which may limit the transferability of the findings to other settings. Second, the study did not include quantitative outcome measures and therefore cannot determine whether the CBPR process led to measurable changes in social isolation, social participation, or well‐being. Third, the voices of socially isolated residents themselves were not fully captured during the preparatory phase because the early participants were mainly healthcare and welfare professionals, arts practitioners, and already active community members. Their perspectives were reflected mainly indirectly through public health nurses, welfare professionals, community volunteers, and family narratives. Fourth, the planned participatory theater workshop was canceled because of the onset of the COVID‐19 pandemic. As a result, this study could not evaluate the implementation, outcomes, or longer‐term effects of the action phase. The findings should therefore be interpreted as a process evaluation of the preparatory or pre‐action phase of a CBPR initiative, rather than as evidence of intervention effectiveness. Future research should examine how socially isolated residents can be involved earlier and more directly while minimizing stigma and pressure, and how digital or hybrid participatory methods might sustain collaboration during crises.

## 5 Conclusions

This CBPR project in Nagi Town illustrates how residents and professionals can collaboratively cultivate dialogue, creativity, and shared ownership in a small rural setting. Although the planned theater workshop was not implemented, the preparatory process itself had practical significance by building relationships, developing a shared vocabulary of equality, and creating a foundation for future collaboration toward social inclusion. These findings suggest that CBPR, particularly when combined with arts‐based engagement, may support collective reflection and relationship building in rural health promotion.

## Author Contributions

**Daisuke Son:** conceptualization, methodology, software, data curation, investigation, validation, formal analysis, project administration, writing – original draft, writing – review and editing. **Akira Matsushita:** conceptualization, methodology, validation, supervision, formal analysis, writing – review and editing. **Michinori Tanaka:** conceptualization, methodology, validation, writing – review and editing. **Mina Ueharu:** conceptualization, methodology, validation, writing – review and editing. **Yusuke Yamauchi:** conceptualization, methodology, validation, writing – review and editing. **Kunihiro Tsujikawa:** conceptualization, methodology, validation, writing – review and editing. **Shoko Uetsuki:** conceptualization, methodology, validation, writing – review and editing. **Naoko Tateishi:** conceptualization, methodology, validation, writing – review and editing. **Masako Nishi:** conceptualization, methodology, validation, writing – review and editing. **Akiko Ichii:** conceptualization, methodology, validation, writing – review and editing. **Suhkye Shin:** conceptualization, methodology, validation, writing – review and editing. **Naoki Sugawara:** conceptualization, methodology, validation, writing – review and editing.

## Funding

The authors have nothing to report.

## Ethics Statement

This study was approved by the Institutional Review Board of the Faculty of Medicine, University of Tokyo (Approval No. 11760). All participants received written and/or oral explanations of the study objectives and procedures and provided informed consent prior to participation. The study was conducted in accordance with the principles of the Declaration of Helsinki.

## Conflicts of Interest

The authors declare no conflicts of interest.

## Data Availability

The data that support the findings of this study are available from the corresponding author upon reasonable request.
